# Sequential non-invasive following short-term invasive mechanical ventilation in the treatment of tuberculosis with respiratory failure: a randomized controlled study

**DOI:** 10.1186/s12890-021-01563-x

**Published:** 2021-06-23

**Authors:** Nai-Min Kang, Nan Zhang, Bao-Jian Luo, En-Dong Wu, Jian-Quan Shi, Liang Li, Li Jiang

**Affiliations:** 1 Department of ICU, Beijing Chest Hospital, Capital Medical University, 9 Beiguan Street, Tongzhou District, Beijing, 101149 China; 2 Department of Intensive Care Unit, Xuanwu Hospital, Capital Medical University, Beijing, 100053 China

**Keywords:** Non-invasive mechanical ventilation, Sequential mechanical ventilation, Pulmonary tuberculosis, Respiratory failure, Randomized controlled study

## Abstract

**Background:**

Invasive and non-invasive mechanical ventilation (MV) have been combined as sequential MV in the treatment of respiratory failure. However, the effectiveness remains unclear. Here, we performed a randomized controlled study to assess the efficacy and safety of sequential MV in the treatment of tuberculosis with respiratory failure.

**Methods:**

Forty-four tuberculosis patients diagnosed with respiratory failure were randomly divided into sequential MV group (n = 24) and conventional MV group (n = 20). Initially, the patients in both groups received invasive positive pressure ventilation. When the patients' conditions were relieved, the ventilation modality in sequential MV group was switched to oronasal face mask continuous positive airway pressure until weaning.

**Results:**

After treatment, the patients in sequential MV group had similar respiratory rate, heart rate, oxygenation index, alveolo-arterial oxygen partial pressure difference (A-aDO_2_), blood pH, PaCO_2_ to those in conventional MV group (all *P* value > 0.05). There was no significant difference in ventilation time and ICU stay between the two groups (*P* > 0.05), but sequential MV group significantly reduced the time of invasive ventilation (mean difference (MD): − 36.2 h, 95% confidence interval (CI) − 53.6, − 18.8 h, *P* < 0.001). Sequential MV group also reduced the incidence of ventilator-associated pneumonia (VAP; relative risk (RR): 0.44, 95% CI 0.24, 0.83, *P* = 0.006) and atelectasis (RR:0.49, 95% CI 0.24,1.00, *P* = 0.040).

**Conclusions:**

Sequential MV was effective in treating tuberculosis with respiratory failure. It showed advantages in reducing invasive ventilation time and ventilator-associated adverse events.

**Registration number for clinical trial:**

Chinese Clinical Trial Registry ChiCTR2000032311, April 21st, 2020

## Background

Pulmonary tuberculosis is a common pulmonary disease in both developed and developing countries. In 2011, 8.7 million new cases of active tuberculosis emerged and 1.4 million cases died worldwide [1]. In 2017, tuberculosis caused 1.6 million global deaths, exceeding deaths caused by any other infectious disease [2]. Patients with active pulmonary tuberculosis often suffer from pulmonary dysfunction due to hemoptysis, interstitial infiltration, caseous pneumonia and other severe complications of tuberculosis, which may probably develop to respiratory failure [3]. Although acute respiratory failure occurs in only 1.5% pulmonary tuberculosis, the mortality often reaches higher than 50% [4].

The most effective way to treat respiratory failure is mechanical ventilation (MV), which supports to relieve symptoms in acute phase and to gain opportunity for the later treatment [5]. The main adverse effects of invasive MV, which usually involves transoral tracheal intubation, include ventilator-associated injury and ventilator-associated infection [6]. Consequently, non-invasive MV has emerged as an optional strategy for patients with respiratory failure due to its fewer complications. It has been broadly applied to patients with severe acute respiratory syndrome (SARS), H1N1, chronic obstructive pulmonary disease (COPD), and coronavirus disease 2019 (COVID-19) [7–9]. Several clinical trials have also attempted to combine both invasive and non-invasive MV as sequential MV in the treatment of COPD [10]. Application of sequential MV reduces intubation time so that it significantly lowers ventilator-associated pneumonia (VAP) and atelectasis. However, the effectiveness of sequential MV for tuberculosis patients with respiratory failure remains unclear. In this study, we performed a randomized controlled study to assess the efficacy and safety of sequential non-invasive MV following short-term invasive MV in the treatment of tuberculosis with respiratory failure.

## Methods

### Participants

The study was prospectively registered at chictr.org.cn-ChiCTR2000032311, and was reported according to CONSORT guidelines. From April 2020 to December 2020, the active tuberculosis patients diagnosed with respiratory failure and treated in ICU of Beijing Chest Hospital, were enrolled in this study. The research protocol was approved by the Human Ethics Committee of Beijing Chest Hospital (2019-clinic-64) and the study was carried out based on the Declaration of Helsinki 1964. Written informed consent was obtained from all participants.

*Included criteria* (1) diagnosed with pulmonary tuberculosis according to medical history, tests for Mycobacterium tuberculosis, and chest radiography, (2) respiratory rate > 30 breaths/min, (3) artery pressure of oxygen (PaO_2_) < 60 mm Hg, (4) arterial carbon dioxide partial pressure (PaCO_2_) > 50 mm Hg, and (5) oxygenation index (PaO_2_/FiO_2_) < 300 mm Hg.

*Exclusion criteria* (1) diagnosed with tuberculosis in central nervous system, (2) with severely damaged lung and probably to be ventilator dependent, (3) with lung cancer or cachexia, (4) acute physiology and chronic health evaluation II (APACHE II) score > 16 [11], (5) with multiple organ dysfunction and prone to be ventilator dependent, (6) with stress ulcer-induced gastrointestinal bleeding or gastrointestinal perforation, and (7) with facial deformity and unable to receive non-invasive MV.

### Treatment protocol

Using computer-generated random numbers, all patients were randomly divided into sequential MV group and conventional MV group. At beginning, patients in both groups received invasive positive pressure ventilation (IPPV) using following ventilation modality: synchronized intermittent mandatory ventilation (SIMV) + pressure support ventilation (PSV) + positive end expiratory pressure (PEEP). Subjects were ventilated with a pressure support level targeting an expired tidal volume of 6–8 mL/kg and a respiratory rate of < 30 breaths/min. FiO_2_ was adjusted to maintain peripheral oxygen saturation (SpO_2_) at > 92% with PEEP of at least 5 cm H_2_O (1 cm H_2_O = 0.098 kPa). SIMV was set at 10–12 breaths/min and PSV was adjusted to 10–12 breaths/min.

When the patients' conditions were relieved, the ventilation modality in sequential MV group was switched to oronasal face mask continuous positive airway pressure (CPAP) until weaning. The indication for extubation included a stable blood pressure with FiO_2_ ≤ 55%, SpO2 ≥ 95%, respiratory rate ≤ 30 breaths/min and tidal volume ≥ 6 mL/kg. The patients in conventional MV group received persistent IPPV until weaning.

During the study, all patients received routine treatment of active tuberculosis and mixed infections with fluid therapy and nutritional supports. The vital signs of patients were carefully monitored during the ventilation and any necessary management was placed for symptomatic treatment.

### Data collection

The basic characteristics of all patients were collected, including: age, sex, body mass index (BMI), APACHE II score, medical history and chest radiography.

The primary end point of this study was assessed by detecting respiratory parameters [including respiratory rate, heart rate, oxygenation index, alveolo-arterial oxygen partial pressure difference (A-aDO_2_), PaCO_2_ and blood pH], and inflammatory parameters [white blood cells (WBC), percentage of neutrophil (NEU) and C-reactive protein (CRP). The respiratory parameters were recorded before treatment (baseline) and in every 8 h during the treatment. And the inflammatory parameters were detected at baseline and in every 24 h. The data in baseline and after treatment (defined as the point when FiO_2_ reduced to 50%) were analyzed and compared between the two groups.

The secondary end-point was evaluated by comparing in-hospital outcomes between the two groups, including ventilation time, invasive mechanical ventilation time, the length of ICU stay, visual analogue scale (VAS) score, total cost (RMB), VAP and atelectasis. VAS score was evaluated by doctors and nurses with a scale of 0–10 (0: no pain, 10: utmost pain), and the mean VAS score during the study was calculated and recorded.

### Statistical analysis

Data analysis was performed using SPSS 23.0 (IBM Corp., Armonk, NY, USA). Continuous variables such as age, BMI, respiratory and inflammatory parameters were presented as mean ± standard deviation (SD). Categorical variables such as sex were presented as number (percentage). Significance was analyzed using student’s *t* test for continuous variables and Chi-square test for categorical variables. As for in-hospital outcomes, mean difference (MD) and relative ratio (RR) with 95% confidence interval (CI) were calculated for continuous variables and categorical variables, respectively. Two-sided *P* value < 0.05 was considered to be statistically significant.

## Results

**Fig. 1:**
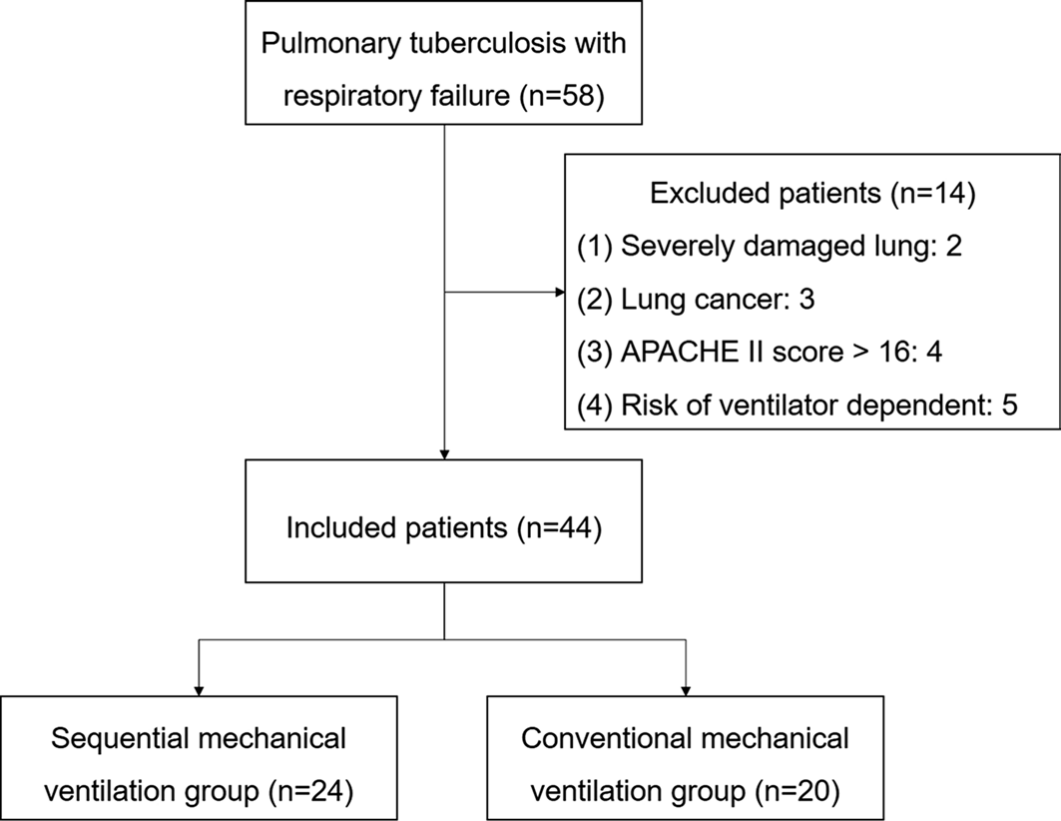
The flow chart of the study. APACHE II: acute physiology and chronic health evaluation II

The flow chart of the study was illustrated in Fig. 1. After inclusion, forty-four patients with 63.6% of male and an age range of 22–88 were randomly divided into sequential MV group (n = 24) and conventional MV group (n = 20). The mean period of tuberculosis in the included participants was 7.1 ± 89 yrs, and no one was diagnosed with extra-pulmonary tuberculosis. Thirteen (29.5%) patients were accompanied with diabetes mellitus, and twenty-six (59.1%) subjects got bacterial infection. At baseline, the mean respiratory rate, heart rate, oxygenation index, A-aDO_2_, PaCO_2_, blood pH, WBC, percentage of NEU and CRP in all included subjects were 36.3 ± 4.6/min, 136.6 ± 11.6/min, 92.5 ± 38.0 mm Hg, 65.3 ± 26.8 mm Hg, 68.7 ± 19.8 mm Hg, 7.27 ± 0.09, 15.8 ± 5.0 × 10^9^/L, 87.9 ± 4.2%, 157.6 ± 46.4 mg/mL, respectively (Table 1).

**Table 1:** Basic characteristics of the included patients before the treatment

Parameters	Total (n = 44)	Sequential MV group (n = 24)	Conventional MV group (n = 20)	*P* value^#^
Age (yrs)	61.3 ± 14.5	63.9 ± 13.0	58.3 ± 15.9	0.209
Male (%)	28 (63.6)	17 (70.8)	11 (55.0)	0.277
BMI (kg/m^2^)	22.4 ± 2.5	22.8 ± 2.0	22.0 ± 3.0	0.307
Period of tuberculosis (yrs)	7.1 ± 8.9	8.4 ± 11.1	5.4 ± 5.2	0.243
Smoking (%)	28 (63.6)	15 (62.5)	13 (65.0)	0.864
Alcohol consuming (%)	23 (52.3)	12 (50.0)	11 (55.0)	0.741
COPD (%)	13 (29.5)	8 (33.3)	5 (25.0)	0.757
Pulmonary cavity (%)	14 (31.8)	7 (29.2)	7 (35.0)	0.679
Diabetes mellitus (%)	13 (29.5)	7 (29.2)	6 (30.0)	0.952
Hypertension (%)	8 (18.2)	6 (25.0)	2 (10.0)	0.199
Bacterial infection (%)	26 (59.1)	14 (58.3)	12 (60.0)	0.911
Application of antibiotic (%)	26 (59.1)	14 (58.3)	12 (60.0)	0.911
APACHE II score	11.4 ± 2.6	12.0 ± 2.3	10.7 ± 2.8	0.109
Respiratory rate (/min)	36.3 ± 4.6	35.7 ± 5.1	37.2 ± 3.9	0.289
Heart rate (/min)	136.6 ± 11.6	134.2 ± 13.8	139.5 ± 7.7	0.134
Oxygenation index (mm Hg)	92.5 ± 38.0	92.0 ± 43.8	93.2 ± 30.8	0.920
A-aDO_2_ (mm Hg)	65.3 ± 26.8	63.0 ± 26.0	68.1 ± 28.3	0.544
PaCO_2_ (mm Hg)	68.7 ± 19.8	66.4 ± 18.2	71.5 ± 21.7	0.402
Blood pH	7.27 ± 0.09	7.30 ± 0.09	7.25 ± 0.09	0.087
WBC (× 10^9^/L)	15.8 ± 5.0	16.4 ± 5.8	15.1 ± 3.7	0.389
Percentage of NEU (%)	87.9 ± 4.2	88.3 ± 5.1	87.4 ± 2.8	0.446
CRP (mg/mL)	157.6 ± 46.4	159.4 ± 48.3	155.4 ± 45.2	0.779

^#^Inter-group difference was calculated between sequential MV group and conventional MV group

*MV* mechanical ventilation, *BMI* body mass index, *COPD* chronic obstructive pulmonary disease, *APACHE* acute physiology and chronic health evaluation, *A-aDO*_*2*_ alveolo-arterial oxygen partial pressure difference, *WBC* white blood cell, *NEU* neutrophil, *CRP* C-reactive protein

**Fig. 2:**
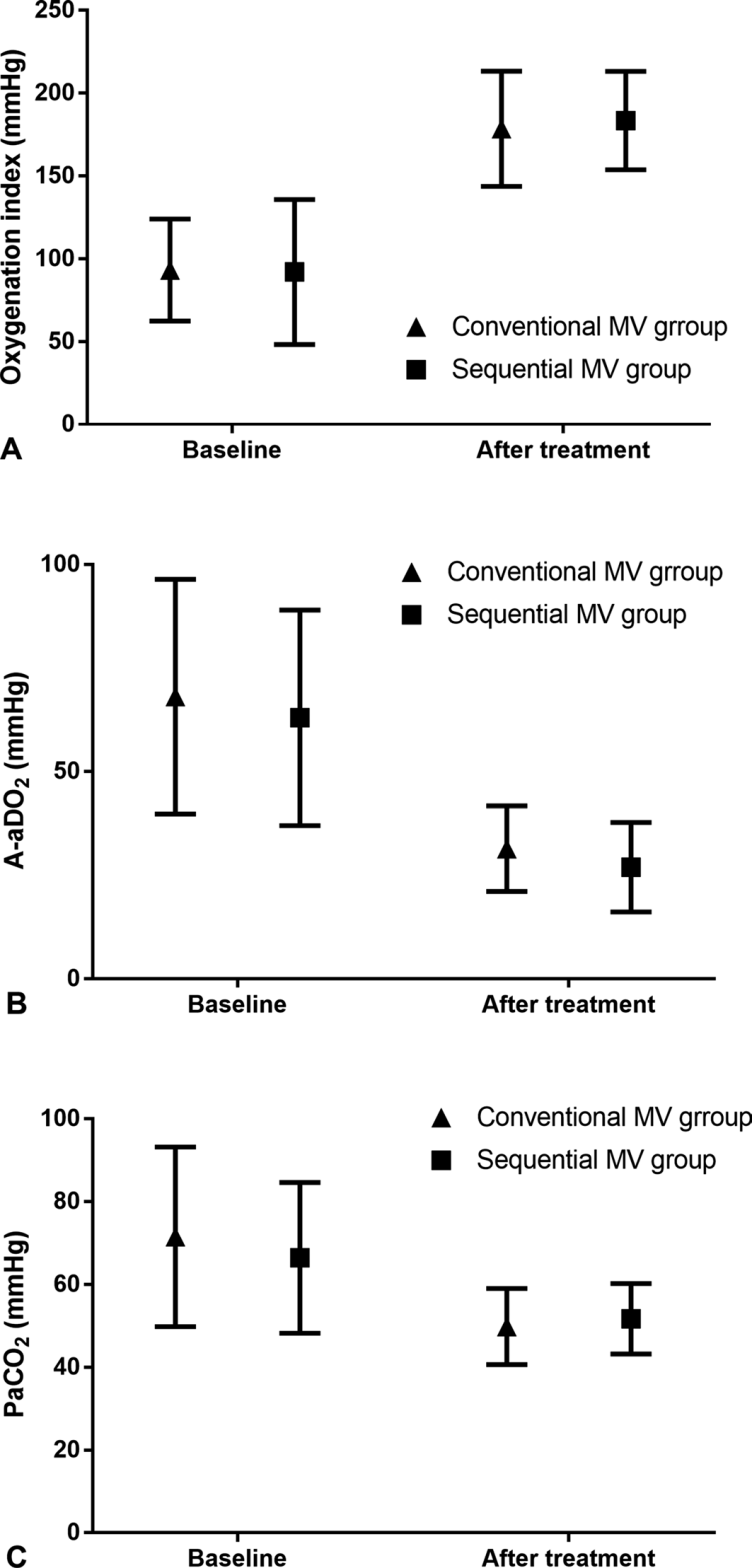
Comparison of oxygenation index (**A**), A-aDO_2_ (**B**), and PaCO_2_ (**C**) between the two groups at baseline and after treatment

The baseline characteristics were similar in conventional MV group and sequential MV group (all *P* value > 0.05). After treatment, all respiratory and inflammatory parameters in each group significantly ameliorated with no any death case occurred. Breathing frequency and heart rate significantly decreased in both sequential MV group and conventional MV group. When FiO_2_ of ventilation reduced to 50%, the patients in sequential MV group had similar respiratory rate, heart rate, oxygenation index, A-aDO_2_, PaCO_2_, blood pH, WBC, percentage of NEU and CRP to those in conventional MV group (all *P* value > 0.05) (Table 2). Correspondingly, the improvement of oxygenation index, A-aDO_2_ and PaCO_2_ in sequential MV group were paralleled to those in conventional MV group (Fig. 2).

**Table 2:** Comparison of clinical outcomes between the two groups after treatment

Parameters	Sequential MV group (n = 24)	Conventional MV group (n = 20)	*P* value
Respiratory rate (/min)	25.7 ± 4.3	25.0 ± 2.7	0.551
Heart rate (/min)	99.1 ± 8.4	98.6 ± 5.4	0.808
Oxygenation index (mm Hg)	183.3 ± 29.7	178.4 ± 34.8	0.618
A-aDO_2_ (mm Hg)	27.0 ± 10.8	31.4 ± 10.3	0.172
PaCO_2_ (mm Hg)	51.7 ± 8.5	49.8 ± 9.2	0.480
Blood pH	7.38 ± 0.05	7.39 ± 0.04	0.412
WBC (× 10^9^/L)	10.2 ± 2.8	9.6 ± 1.7	0.390
Percentage of NEU (%)	79.3 ± 5.7	77.9 ± 3.8	0.342
CRP (mg/mL)	67.0 ± 36.6	66.0 ± 35.5	0.924

*MV* mechanical ventilation, *A-aDO*_*2*_ alveolo-arterial oxygen partial pressure difference, *WBC* white blood cell, *NEU* neutrophil, *CRP* C-reactive protein

There was no significant difference in total ventilation time and the length of ICU stay between the two groups (*P* > 0.05), but sequential MV group significantly reduced the time of invasive ventilation (50.8 ± 25.3 h vs. 87.0 ± 32.3 h; MD: − 36.2 h, 95% CI − 53.6, − 18.8 h, *P* < 0.001) (Table 3). Sequential MV group also dramatically reduced the incidence of VAP (33.3% vs. 75.0%; RR: 0.44, 95% CI 0.24, 0.83, *P* = 0.006) and atelectasis (29.2% vs. 60.0%; RR: 0.49, 95% CI 0.24, 1.00, *P* = 0.040) compared with conventional MV group. Patients in sequential MV group were much more comfortable with lower VAS score (6.5 ± 1.1 vs. 7.7 ± 1.1; MD: − 1.2, 95% CI − 1.9, − 0.6, *P* < 0.001). Additionally, in-hospital cost was statistically lower in sequential MV group than in conventional MV group (50 ± 22 thousand vs. 63 ± 20 thousand; MD: − 13 thousand, 95% CI − 25, − 1 thousand, *P* = 0.045).

**Table 3:** Comparison of in-hospital outcomes between the two groups

Parameters	Sequential MV group (n = 24)	Conventional MV group (n = 20)	MD or RR	95% CI	*P* value
Ventilation time (h)	87.7 ± 36.0	87.0 ± 32.3	0.7	− 19.5, 20.9	0.946
Invasive mechanical ventilation time (h)	50.8 ± 25.3	87.0 ± 32.3	− 36.2	− 53.6, − 18.8	< 0.001
VAP (%)	8 (33.3)	15 (75.0)	0.44	0.24, 0.83	0.006
Atelectasis (%)	7 (29.2)	12 (60.0)	0.49	0.24, 1.00	0.040
ICU stay (d)	5.5 ± 2.6	4.4 ± 1.4	1.1	− 0.1, 2.3	0.086
VAS score	6.5 ± 1.1	7.7 ± 1.1	− 1.2	− 1.9, − 0.6	< 0.001
Total cost (thousand RMB)	50 ± 22	63 ± 20	− 13	− 25, − 1	0.045

*MV* mechanical ventilation, *MD* mean difference, *RR* relative risk, *CI* confidence interval, *VAP* ventilator-associated pneumonia, *VAS* visual analogue scale

## Discussion

The present study showed sequential MV was an effective strategy to reverse respiratory function in tuberculosis patients and was comparable to conventional MV in improving oxygenation index and A-aDO_2_. Our results were consistent with some former studies. Frat et al. [12] evaluated the clinical efficacy of humidified oxygen using sequential MV (which included high-flow nasal cannula alternated with non-invasive MV) in acute hypoxemic respiratory failure. The results showed better improvement in oxygenation and tachypnea in sequential MV compared to standard oxygen therapy. Burns et al. [13] reviewed relevant randomized controlled trials (RCT) to explore the efficacy of non-invasive MV as a weaning strategy for ventilation in patients with respiratory failure. Compared to continued invasive MV, non-invasive weaning reduced the mortality and the incidence of pneumonia, without increasing the risk of weaning failure or reintubation. Osadnik et al. [14] performed another systemic review to compare the efficacy of non-invasive MV in conjunction with usual care versus usual care without MV in acute hypercapnic respiratory failure. Their results showed that non-invasive MV was beneficial to reducing the mortality and endotracheal intubation, and it could be regarded as a first-line intervention in conjunction with usual care for patients with respiratory failure. All evidence proved the non-invasive MV as an efficient weaning strategy for ventilation.

Although there was no significant difference in ventilation time and the length of ICU stay between the two groups, our study detected sequential MV was able to reduce the incidence of VAP and atelectasis, mainly due to the reduced time of invasive ventilation. A recent meta-analysis performed by Huang et al. also assessed the safety of sequential MV versus conventional MV in the treatment of acute exacerbation of COPD (AECOPD) [15]. Their results showed the application of sequential MV at the pulmonary infection control window significantly reduced VAP incidence (RR: 0.20, 95% CI 0.16–0.26), mortality (RR: 0.38, 95% CI 0.26–0.55), reintubation rate (RR: 0.39, 95% CI 0.27–0.55), invasive ventilation time (MD: − 9.23, 95% CI − 10.65, − 7.82), total ventilation time (MD: − 4.91, 95% CI − 5.99, − 3.83), and the length of ICU stay (MD: − 5.10, 95% CI − 5.43, − 4.76). Poor tolerance to non-invasive MV accounted for 5–25% intubation in the hypoxemic patients [16–19]. Our data showed the patients in sequential MV group were less painful than those in conventional MV, representing a better tolerance of non-invasive MV than invasive MV. However, some research reported a reversed result. Frat et al. [12] showed high-flow nasal cannula was better tolerated than non-invasive MV with a lower VAS score. Moreover, we found sequential MV was a cost-effective strategy, which was also confirmed by other investigations [20]. These advantages in comfort and economy will highly improve the acceptance of sequential MV by patients.

It has been discussed that non-invasive MV is not suitable for patients with severe bronchial infections and heavy sputum, since they are unable to cough and unconscious to severe hypercapnia. In these conditions, invasive MV with intubation is usually needed to facilitate sputum drainage and improve respiratory function. During invasive MV, however, it is more likely to get repeated VAP due to implementation of the artificial airway [21, 22]. Once VAP occurs, the patients’ condition usually gets worse, and weaning of ventilation is difficult to be performed [23, 24]. Therefore, the key to reduce the incidence of VAP is to shorten the intubation time or remove the implementation. We previously found fiber optic bronchoscope (FOB) was not only useful in the diagnosis of suspected pneumonia, but could be also applied to locate and suction sputum [25]. Song et al. [26] reported the application of FOB was effective and safe in AECOPD during weaning of sequential MV, which decreased the total ventilation time, the length of ICU stay, reintubation rate, incidence of VAP. The application of FOB may further improve the tolerance of non-invasive MV.

The limitations of the present study should be also noted. First, it was designed as a pilot study with a relatively small sample size. Only 44 patients were included in this study and further study is needed to explore the application of sequential MV with bigger sample size. Second, we only included patients with APACHE II score ≤ 16 and patients with severe chronic diseases were excluded. Hence, we are unable to estimate the real mortality. Third, we have only provided data to compare the short-term clinical outcomes between the two groups, but we failed to assess the mid-term or long-term efficacy and safety by follow-up. Fourth, VAS score was evaluated subjectively, though the nurses and doctors were trained to follow to standard criteria. Fifth, we failed to present the temporal trends of the various respiratory parameters for each ICU day, since these data could not be directly compared between the two groups. Sixth, multivariate analyses were not performed in this study since we only included 44 patients, and the results could be affected by some patient characteristics or co-morbid conditions they had. Seventh, this was an open trial, as blinding to type of ventilation is not possible. In addition, it may be not suitable to compare our data with former studies, since most of them focused on patients diagnosed with COPD. Finally, we failed to detect the best time point for switching from invasive MV to non-invasive MV, which is important for clinical work and therefore badly needed for further investigations.

## Conclusions

Sequential MV was safe and effective to improve respiratory function with advantages in reducing invasive ventilation time and ventilator-associated adverse events. Sequential MV can be regarded as an optional strategy to treat tuberculosis patients with respiratory failure.

## Data Availability

The datasets used and/or analyzed during the current study are available from the corresponding author on reasonable request.

## Abbreviations

MV: Mechanical ventilation
IPPV: Invasive positive pressure ventilation
CPAP: Continuous positive airway pressure
A-aDO_2_: Alveolo-arterial oxygen partial pressure difference
MD: Mean difference
CI: Confidence interval
VAP: Ventilator-associated pneumonia
RR: Relative risk
SARS: Severe acute respiratory syndrome
COPD: Chronic obstructive pulmonary disease
